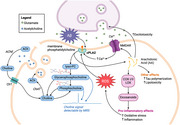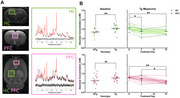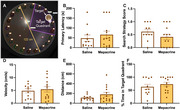# Exploring the Effects of Phospholipase A2 Inhibition on Brain Metabolism and Pathology in a Rat Model of Alzheimer’s Disease

**DOI:** 10.1002/alz70859_103238

**Published:** 2025-12-25

**Authors:** Emily Hiles, Colleen Bailey, Wendy Oakden, Tina Beckett, Mary Hill, JoAnne McLaurin, Jamie Near

**Affiliations:** ^1^ University of Toronto, Toronto, ON Canada; ^2^ Sunnybrook Research Institute, Toronto, ON Canada; ^3^ Biological Sciences, Sunnybrook Research Institute, Toronto, ON Canada

## Abstract

**Background:**

Magnetic resonance spectroscopy (MRS) offers a non‐invasive modality to explore metabolic changes associated with Alzheimer’s disease (AD) pathogenesis and treatment efficacy. Studies in both humans and animal models have observed elevated choline in AD which may suggest an increase in cell membrane breakdown by phospholipase A2 (PLA2). PLA2 has also been linked to multiple hallmarks of AD pathology such as cognitive decline and inflammation (Figure 1). We hypothesized that PLA2 inhibition will reduce elevated choline levels and improve AD pathology.

**Method:**

TgF344‐AD rats (Tg) and non‐transgenic (NTg) litter mates at 14 months old (N=50) were treated via daily IP injections with either 5mg/kg mepacrine (a global PLA2 inhibitor) or saline for one week followed by injections every other day for two weeks. Metabolite concentrations were measured in the right dorsal hippocampus (HC) and pre‐frontal cortex (PFC) using localized proton MRS (Figure 2A) at baseline, 7 days, and 21 days. After 7 days of treatment, animals completed the Barnes maze including acquisition training, long‐term memory probe (Figure 3A), and reversal training. At day 21, animals were sacrificed, tissues were collected, and brain sections were stained with 6F3D, PHF1, IBA1, and GFAP to observe changes to hippocampal pathology with PLA2 inhibition.

**Result:**

At baseline, choline concentration was significantly elevated in the HC (6.8±1.9% increase, P=0.0012) but not the PFC (3.4±2.3% increase, P=0.1426; Figure 2B) of Tg animals. These levels were significantly reduced at day 7 and 21 of treatment in the HC of mepacrine treated Tg animals (6.6±1.9% decrease, P=0.0141; 6.9±1.3% decrease, P=0.0012; Figure 2B). In the PFC of mepacrine treated Tg animals, choline levels were only significantly reduced by day 21 of treatment (8.1±1.8% decrease, P=0.0027; Figure 2B). Contrary to our hypothesis, no significant differences were observed between saline and mepacrine treated Tg animals in any of the parameters measured in the probe trail (Figure 3B‐F). Pathology data is currently being analyzed.

**Conclusion:**

These findings indicate choline concentration is associated with PLA2 activity in AD. However, changes to spatial memory formation with PLA2 inhibition were not observed. This is contrary to the literature; thus, further research is needed to understand if PLA2 plays a role in AD associated memory impairment.